# From animal models to human disease: a genetic approach for personalized medicine in ALS

**DOI:** 10.1186/s40478-016-0340-5

**Published:** 2016-07-11

**Authors:** Vincent Picher-Martel, Paul N. Valdmanis, Peter V. Gould, Jean-Pierre Julien, Nicolas Dupré

**Affiliations:** Department of Psychiatry and Neuroscience, Research Centre of Institut Universitaire en Santé Mentale de Québec, Laval University, 2601 Chemin de la Canardière, Québec, QC G1J 2G3 Canada; Departments of Pediatrics and Genetics, Stanford University, 269 Campus Drive, CCSR 2110, Stanford, CA 94305-5164 USA; Division of Anatomic Pathology and Neuropathology, Department of Medical Biology, CHU de Québec, Hôpital de l’Enfant-Jésus, 1401, 18th street, Québec, QC Canada G1J 1Z4; Axe Neurosciences & The Department of Medicine, Faculty of Medicine, CHU de Québec, Laval University, 1401, 18th street, Québec, QC G1J 1Z4 Canada

**Keywords:** Amyotrophic lateral sclerosis (ALS), Personalized medicine, Animal models, Mouse, Gene therapy, Biomarkers, Frontotemporal dementia (FTD)

## Abstract

Amyotrophic Lateral Sclerosis (ALS) is the most frequent motor neuron disease in adults. Classical ALS is characterized by the death of upper and lower motor neurons leading to progressive paralysis. Approximately 10 % of ALS patients have familial form of the disease. Numerous different gene mutations have been found in familial cases of ALS, such as mutations in *superoxide dismutase 1 (SOD1)*, *TAR DNA-binding protein 43 (TDP-43)*, *fused in sarcoma (FUS)*, *C9ORF72, ubiquilin-2 (UBQLN2)*, *optineurin (OPTN)* and others. Multiple animal models were generated to mimic the disease and to test future treatments. However, no animal model fully replicates the spectrum of phenotypes in the human disease and it is difficult to assess how a therapeutic effect in disease models can predict efficacy in humans. Importantly, the genetic and phenotypic heterogeneity of ALS leads to a variety of responses to similar treatment regimens. From this has emerged the concept of personalized medicine (PM), which is a medical scheme that combines study of genetic, environmental and clinical diagnostic testing, including biomarkers, to individualized patient care. In this perspective, we used subgroups of specific ALS-linked gene mutations to go through existing animal models and to provide a comprehensive profile of the differences and similarities between animal models of disease and human disease. Finally, we reviewed application of biomarkers and gene therapies relevant in personalized medicine approach. For instance, this includes viral delivering of antisense oligonucleotide and small interfering RNA in SOD1, TDP-43 and C9orf72 mice models. Promising gene therapies raised possibilities for treating differently the major mutations in familial ALS cases.

## Introduction

**Fig. 1:**
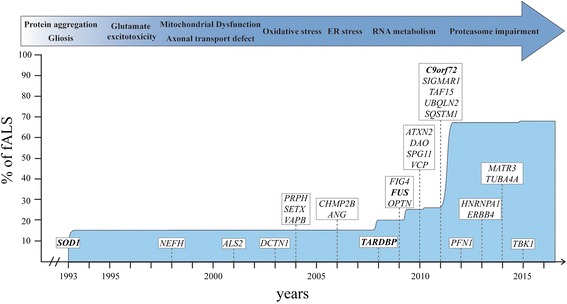
Timeline of gene discovery and pathogenic mechanisms in ALS. Schematic representation of years of discovery of most important genes implicated in ALS. Mutation in the superoxide dismutase 1 (*SOD1*) represent approximately 15 % of familial ALS cases (fALS), mutation in *C9orf72* represent 35-40 % and both TAR-DNA-binding protein (*TARDBP*) and Fused in sarcoma (*FUS*) for 4 % each. Other genes represent less than 1 % each. Protein aggregation and gliosis are pathological hallmark of ALS and have been discovered before the beginning of the illustrated timeline. ER endoplasmic reticulum; *NEFH* neurofilament heavy; *ALS2* alsin; *DCTN1* dynactin; *PRPH* peripherin; *SETX* senataxin; *VAPB* vesicle-associated membrane protein-associated protein B; *CHMP2B* Charged multivesicular body protein 2B; *ANG* angiogenin; *FIG4* phosphoinositide 5-phosphatase; *OPTN* optineurin; *ATXN2* ataxin 2; *DAO* D-amino acid oxidase; *SPG11* spastic paraplegia 11; *VCP* valosin containing protein; *SIGMAR1* sigma non-opioid intracellular receptor 1; *TAF15* TATA-box binding protein associated factor 15; *UBQLN2* ubiquilin-2; *SQSTM1* sequestosome 1; *PFN1* profilin-1; *HNRNPA1* heterogeneous nuclear ribonucleoprotein A1; *ERBB4* erb-2 receptor tyrosine kinase 4; *MATR3* matrin 3; *TUBA4A* tubulin alpha-4a; *TBK1* TANK-binding kinase 1

Amyotrophic Lateral Sclerosis (ALS) is the most common motor neuron disorder in adults. It is characterized by progressive death of upper and lower motor neurons. This degeneration leads to paralysis and to patient death within 2 to 5 years after disease onset. In the last ten years, a wide variety of gene mutations have been discovered for the familial form of the disease (fALS), leading to an impressive genetic heterogeneity. Expanded hexanucleotide repeats in *C9orf72* account for nearly 35 % of familial cases, mutations in superoxide dismutase 1 (*SOD1*) for 20 %, mutations in TAR DNA-binding protein (*TARDBP*) encoding TDP-43 and fused in sarcoma (*FUS*) for 4 % each. Other genes like p62 (*SQSTM1*), Ubiliquin-2 (*UBQLN2*), TANK-binding kinase 1 (*TBK1*) and Optineurin (*OPTN*) account for less than 1 % each [1] (Fig. 1). Genetic heterogeneity and other unknown causes of the sporadic form of ALS (sALS) lead to a phenotypic variability which increases treatment complexity of the disease. Numerous pathological cellular mechanisms are identified in ALS and have been recently reviewed [2]. This includes oxidative stress, mitochondrial defect, axonal transport impairment, protein aggregation, excitotoxicity, endoplasmic reticulum stress, abnormal RNA processing and neuroinflammation with a role of non-neuronal cells (Fig. 1). These mechanisms will not be further discussed in this review.

Development of transgenic animal models is the primary stage for understanding pathophysiology and for testing future effective therapies [3]. The broad variety of genetic mutations identified in ALS and ALS with frontotemporal dementia (ALS-FTD) leads to extensive literature about ALS animal models. Some of these models may raise concerns about their validity for human disease because of their incomplete or differing phenotypes and the lack of treatment reproducibility in humans. In this review, we summarize ALS animal models in mice, rats, fruit flies, worms, zebrafish, dogs and pigs. We compare each model to the clinical presentation of ALS-FTD and discuss the relevance of these models. We also discuss therapies which can be applied to specific gene mutation in a perspective of personalized medicine. For the moment, Riluzole is the only drug approved for ALS treatment. Numerous other drugs established to be efficient in mice have failed in clinical trials [4]. Interestingly, many of these drugs were effective in a small proportion of patients but were unable to exhibit a favorable effect on overall trial. These curious results are probably caused by the high variability in clinical phenotypes and biological pathways found in ALS and underscore the need for evaluating a personalized medicine approach to treatment.

## Review

### Clinical manifestation and epidemiology of amyotrophic lateral sclerosis

#### Classical ALS

**Fig. 2:**
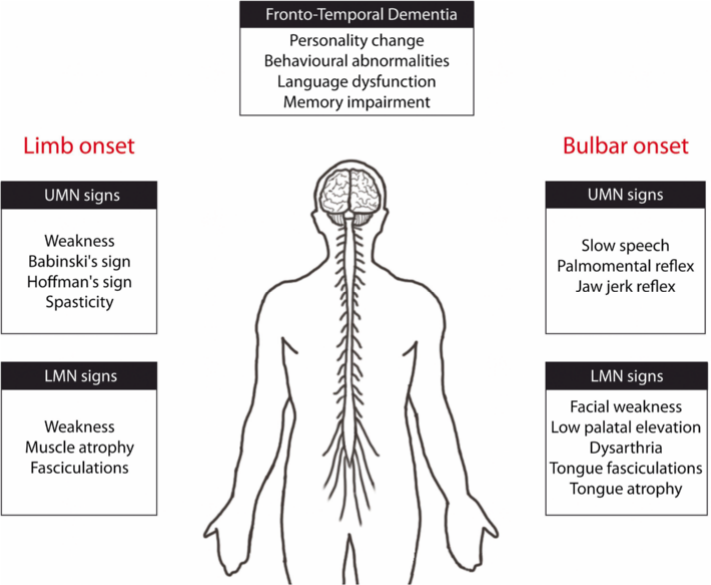
Clinical findings in Amyotrophic lateral sclerosis (ALS). Signs and symptoms are divided by affected motor neuron. Both upper motor neurons (UMN) and lower motor neurons (LMN) have to be affected for the diagnosis of ALS. Different combination of LMN and UMN signs can be observed. Limb onset is found in around 65 % of patients but most patients will develop signs in both bulbar region and limbs within the course of disease. Up to 50 % of ALS patients may have symptoms of Fronto-Temporal dementia

ALS affects both upper (UMN) and lower motor neurons (LMN). It has a wide phenotypic variability determined primarily by three main features: site of onset, rate of progression and relative number of UMN and LMN deficits [5]. Classical spinal onset generally starts with an asymmetric weakness in a limb and the patient will consult a physician because of an unexplained foot drop with or without falls [6]. Weakness will progressively spread to other contiguous limb regions and finally reach respiratory muscles after a few months [5]. Clinical examinations could reveal lower motor neurons signs which include weakness, muscle atrophy, fasciculations and reduced reflexes, whereas upper motor neurons signs include hypertonia, hyperreflexia, Babinski’s sign and Hoffman’s sign [7] (Fig. 2). The onset of symptoms is gradual. It is uncommon for patients to complain about sensory deficit or paresthesia. However, electrophysiological and pathological studies confirmed that response to stimuli can be altered and a loss of large caliber axon was observed [8, 9]. Furthermore, a combination of spinal diffusion tensor imaging (DTI) and electrophysiological recordings demonstrated a subclinical sensory deficit in 85 % of patients with ALS [10].

#### Bulbar ALS

Approximately 20 to 30 % of ALS starts with bulbar onset. Lower motor neuron sign in bulbar onset include facial weakness, low palatal elevation, dysarthria, tongue fasciculations and atrophy. Upper motor neuron signs include slow speech, slow tongue movement, palmomental reflex and jaw jerk reflex [11] (Fig. 2). Change in phonation and uncontrolled crying and laughing, known as pseudobulbar affect, can be present [12]. A differential diagnosis has to be made with progressive bulbar palsy, which is characterized by dysphagia and dysarthria with predominant lower motor involvement. ALS prognosis is generally worse with bulbar onset because of the early respiratory dysfunction.

#### Fronto-temporal dementia

Cognitive impairment in ALS patients frequently occurs in the evolution of disease. These symptoms range from small cognitive impairment (50 %) to official diagnosis of fronto-temporal dementia (FTD). FTD, in up to 15 % of ALS patients, generally presents with personality change, behavioural abnormalities, language dysfunctions and memory impairment [13]. FTD is the second most common form of dementia after Alzheimer’s disease. It is characterized by progressive degeneration of frontal and anterior temporal lobes. Both ALS and FTD share common pathological mechanisms such as TDP-43 cytosolic aggregation [14]. Furthermore, recent findings suggest that a single genetic mutation can cause both diseases, together or separately. C9orf72 and TDP-43 are strongly linked to both disorders whereas SOD1 is rarely mutated in FTD cases [15].

#### Epidemiology

The incidence of amyotrophic lateral sclerosis is 1 to 3 per 100,000 person/years and is relatively similar between countries [16–18]. Usually men have a higher risk (1.2 to 1.5) of developing ALS during their lifetime [19]. The lifetime risk for men is approximately 1:350 and 1:400 for women [20]. Age at onset differs between familial and sporadic forms of ALS. Patients with affected relatives normally develop the disease in their forties or early fifties whereas sporadic cases on average develop it in the late fifties [21–23]. There is also a peak of incidence in the sporadic form between 70-79 years.

Several risk factors for ALS are proposed but none of them have clearly established pathophysiology. Men who served in the military are at higher risk (RR = 1.53; 95 % CI: 1,12 to 2.09) of developing ALS without any regard to war and service [24]. Incidence is also higher among football players (40 fold) and soccer players (6.5 fold) [25, 26]. Some studies have suggested a possible association between physical activity and development of ALS but more work has to be done to confirm this hypothesis [27]. Additionally, head traumas, which persist as major health concern among football and other collision sports players, are being studied as risk factors to ALS. Neuropathology studies described TDP-43 proteinopathy in brains and spinal cords of athletes with ALS-FTD [28]. Finally, lower body mass index and smoking are linked to a higher risk of ALS [29, 30].

#### Neuropathology

Charcot first described the neuropathological features of ALS which consist of muscle atrophy, loss of anterior horn cells and sclerosis of the spinal cord lateral columns. Degeneration affects most of the motor neuron system. Nevertheless, the nuclei controlling eye movement (oculomotor, trochlear and abducens) and fecal and urinary continence (Onufrowicz’s) are generally intact [31]. Motor neurons show histopathological feature such as cytosolic inclusions. Mutation specific cellular structures will be detailed in the following sections. No macroscopic changes are observed in the brain of most ALS patients [32]. However, atrophy of the frontal and temporal cortex including reduced white matter volume can be observed with magnetic resonance imaging (MRI) in ALS-FTD [33, 34]. Combined techniques of voxel-based morphometry (VBM) and MRI demonstrated white matter deficits along corticospinal tract, corpus callosum, cerebellum and in frontal and occipital subcortical regions [35].

Astrocytes and microglia activation, defined as gliosis, is a pathological hallmark of ALS. Analysis from sporadic and familial ALS revealed microglial activation, reactive astrocytes and T cell infiltration in the in spinal cord [36, 37]. Positron emission tomography has brought more evidence of activated microglia in brain of ALS patients. Activated microglia was found in the motor cortex, pons, dorsolateral prefrontal cortex and thalamus. The authors also described a positive correlation between microglia activation in motor cortex and amount of upper motor neuron signs [38].

#### Diagnostic criteria

The El Escorial criteria (EEC) have been employed since 1990 for the diagnosis of ALS and have been revised in 2000 [39]. Diagnosis is based upon UMN signs, LMN signs, the identified gene mutation, electrophysiological and neuroimaging studies, but no definitive diagnostic test are suitable for ALS. For definite ALS, UMN and LMN clinical signs in three different regions are needed. However, EEC is excessively restrictive as some patients are dying without meeting criteria for definite ALS [40]. For example, a patient without an identified gene mutation and with only LMN signs will be classified as clinically suspected ALS or probable ALS with laboratory supports, if present. More recently, the Awaji-Shima criteria was introduced to improve the sensitivity of ALS diagnosis and increases potential entry into clinical trials [41]. Diagnosis of definite ALS required clinical or electrophysiological evidence of UMN and LMN features in one bulbar region and two spinal regions or in three spinal regions. Consequently, ALS can be detected with electrophysiological evidence in clinically unaffected regions of an early stage patient. The most frequent electrophysiological evidences in ALS are fasciculation, fibrillation potentials, positive sharp waves and polyphasic units. A meta-analysis revealed that Awaji-Shima criteria have a sensitivity of 81.1 % as compared to 62.2 % for EEC and Awaji criteria are also better for diagnostic of bulbar onset [42].

### Mutation in familial cases of ALS

#### Superoxide dismutase 1 (SOD1)

##### Specific disease characteristics in humans

Mutations in the *SOD1* gene have been the first described cause of familial ALS [43]. Since 1993, more than 150 missense mutations have been described in *SOD1*, all updated on the ALSoD website (http://alsod.iop.kcl.ac.uk/). Unfortunately, individual mutations are poorly correlated with clinical presentation. Furthermore, the clinical phenotype is often variable between members of the same family. Almost all clinical manifestations of ALS can be observed with *SOD1* mutations [23, 44–47]. However, some authors have stated that the *SOD1* A4V mutation leads to a rapid death after only one year of symptoms [22] and that people with D90A mutations have slow disease progression [45]. Some specific characteristics for *SOD1* mutations are listed in Table 2. Age at onset can vary from 6 to 94 years old with a mean of 40 ± 9.9 to 58.9 ± 12.6 according to studies. Progression of the disease can also vary from 8 months to 18.7 ± 11.4 years. Most of the time, familial *SOD1* ALS starts with an asymmetric weakness in a limb with predominantly lower motor neurons signs. Patients usually suffer from weakness, atrophy, fasciculation and reflexes can be either increased or decreased. Babinski’s sign is often absent. Cognitive symptoms are usually not present in familial SOD1 cases. However, FTD has been described with four *SOD1* mutations, G41S, L144F, I113T and G141X [48–51]. Non-motor symptoms have been described mainly with the D90A mutation, such as urinary symptoms, painful muscle spasm, cerebellar ataxia and sensory symptoms [45, 52].

Neuropathological findings described in patients with *SOD1* mutations include several classes of cytoplasmic inclusions. Lewy-body like hyaline inclusions (LBHI) are the most described inclusions in *SOD1* variants. They have been found in patients with A4V [53], G37R [54], H46R [55], H48Q [56], I113T [57] and L126S [58] mutations. LBHI, in hematoxylin and eosin (H&E) staining, exhibits dense cores with paler peripheral halos. These inclusions are usually composed of granule-coated fibrils, mutated and wild-type SOD1 and ubiquitin [59]. Other bodies, like intracytoplasmic SOD1 inclusions, skein-like inclusion or neurofibrillary tangle, were found in SOD1 familial cases (Table 2). Hyaline inclusions are also present in astrocytes [60].

##### Mouse models

The first animal model carrying a mutation in human *SOD1* was created in 1994 [61]. SOD1^G93A^ was expressed under the control of the human *SOD1* promoter. These mice reproduced most of the clinical and neuropathological findings of ALS. They developed motor deficits in Rotarod and Hangwire tests at 80-90 days and died at 130 days after loss of muscle innervation and motor neuron degeneration. Reports have demonstrated degeneration of the neuromuscular junction before the onset of symptoms, around 40 to 50 days [62, 63]. Gliosis was found before onset and increased in intensity over time [64]. Also, one report has showed an increased proportion of activated microglia from 80 days of age [65] and more recently, positron emission tomography (PET) imaging in the SOD1^G93A^ mouse showed increased inflammation by 110 ± 33 % in the whole brain [66].

It was originally proposed that the pathological effect of SOD1 in ALS was caused by a loss of dismutase function. However, *Sod1* knock-out (KO) mice did not develop ALS up to six months of age [67]. Further studies suggest that *Sod1* KO mice develop a significant distal motor axonopathy without any motor neuron loss [68]. Indeed, the role of SOD1^WT^ in ALS pathology has to be clarified. Some studies have demonstrated that SOD1^WT^ was not implicated in neurodegeneration of ALS. Mice expressing wild-type human SOD1 developed vacuolization of mitochondria and axonal degeneration in the spinocerebellar tract but without motor neuron loss before 2 years of age [69]. However, co-overexpression of SOD1^WT^ in fALS-mutant mice succeeded to increase the ALS phenotype and to convert unaffected mice to ALS [69, 70]. More recently, expression of SOD1^WT^ at same level as seen in SOD1^G93A^ mouse caused an ALS-like phenotype with similar neuropathological findings [71].

**Fig. 3:**
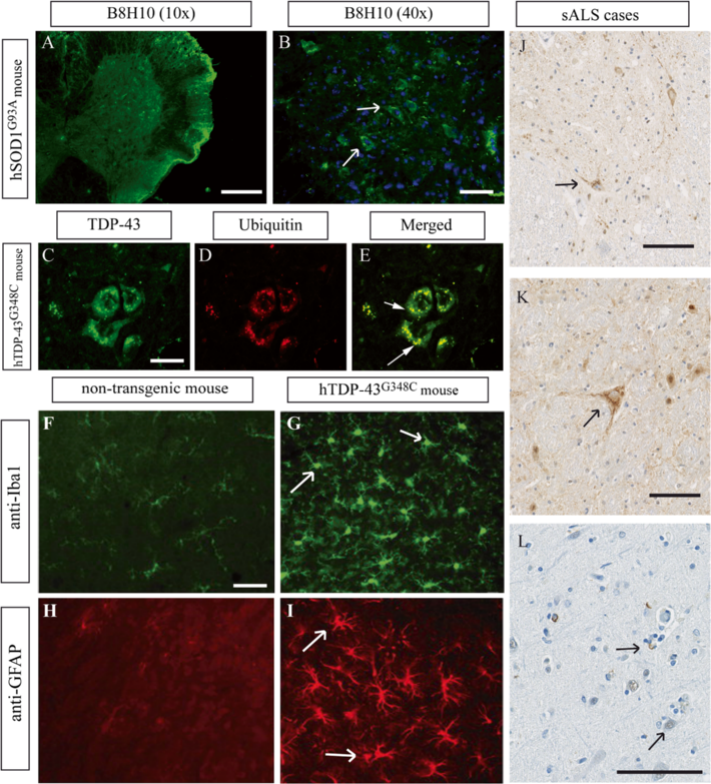
Neuropathological findings in human sALS cases and animal models of ALS. Microscopic pictures of neuropathological findings in ALS models. Our previously published TDP-43 and SOD1 mouse models were exploited for illustration of TDP-43 and SOD1 aggregates with permission. **a** Immunofluorescence microscopy of a hSOD1^G93A^ mouse spinal cord. The B8H10 antibody was utilized for the specific signal of misfolded SOD1. Pictures were taken at 10x and **b** 40x magnification for better visualization of aggregates. **c**-**e** Double immunofluorescence microscopy of 10 months-old a hTDP-43^G348C^ mouse spinal cord using hTDP-43 monoclonal antibody and **d** ubiquitin antibody [138]. Ubiquinated TDP-43 cytoplasmic aggregates can be observed and are typical neuropathological findings in human ALS. **f**-**i** Immunofluorescence of 10 months-old hTDP-43^G348C^ and non-transgenic mice spinal cord. Iba1 antibody **f**-**g** and GFAP antibody **h**-**i** showed increased microgliosis and astrogliosis in a 10 months-old hTDP-43^G348C^ mouse. **j**-**l** Immunohistochemistry of two human sporadic ALS cases using TDP-43 antibody to illustrated typical neuronal cytoplasmic TDP-43 inclusions in lumbar spinal cord (**j**), medulla (**k**) and motor cortex (**l**). Scale bar = 250 μm (**a**); 50 μm (**b**, **f**-**i**); 25 μm (**c**-**e**); 100 μm (**j**-**l**)

Numerous *SOD1* mouse models have been created and they have variable phenotypes, age of disease onset and survival (Table 3). These heterogeneous phenotypes seems to be dependent on specific mutations, expression levels of mutant *SOD1*, gender and genetic background [72]. Generally, females experienced delayed onset and prolonged survival as compared to males and such differences can also be observed in humans [73]. Most *SOD1* mutant mice represent human *SOD1* pathology quite well. Mice develop fatal paralysis with motor neuron deficit, gliosis and intracytoplasmic ubiquitinated *SOD1* inclusions (Fig. 3). However, poor correlation can be made between age of onset and progression in mouse and human. For example, the A4V mutation, the most frequent mutation in humans which causes a rapid disease, is not pathogenic in mouse before 85 weeks [74]. Moreover, the SOD1 G93A mutation, which leads to an early onset and fast progression in mouse, has a slow rate of progression in human (Tables 1 and 2). Interestingly, the D90A mutation in mice and humans shows strong similarities. Homozygous SOD1^D90A^ mice exhibit a slow disease progression and bladder disturbance, which are also found in humans with the same homozygous mutation [45, 75]. Cognitive symptoms have been described in some mouse models. Mice exhibiting the G37R mutation have learning deficits in passive avoidance from 8 months of age and pre-symptomatic SOD1^G93A^ mice exhibit learning delay and long-term memory deficits [76, 77]. Non-motor features such as sensory deficits are described in G37R and D83G [78].

**Table 1:** Clinical characteristics of human SOD1 mutations reproduced in animal models

Mutation	Mean age at onset (y ± SD)	Site at onset	Survival (yr ± SD)	Clinical manifestations				References
				UMN	LMN	Cognitive symptoms	Neuropathological findings	
A4V	47.8 ± 13.3	Limbs	1.4 ± 0,9	Mild	Y, Pred	N	LBHI, NCI	[53, 232, 233]
G37R	40.0 ± 9.9	Limbs	18.7 ± 11.4	Y	Y	N	LBHI	[22, 43, 54]
H46R	49.6 ± 10.9	Legs	17.3 ± 10.7	Y	Y, Pred	N	LBHI	[55, 234]
H48Q	54	nd	8 months	nd	Y, Pred	nd	LBHI,SLI	[56, 235]
L84V	53.8 ± 15.3	Arms	1.6 ± 0.5	Y	Y, Pred	N	nd	[236, 237]
D83G	55	Legs	6	Y	Y, Pred	N	nd	[46]
G85R	nd	nd	nd	nd	nd	nd	nd	[43]
D90A	44	Legs	13	Y	Y	nd	nd	[45, 52]
G93A	47.4 ± 12.4	Limbs	10.0 ± 6.2	nd	nd	N	nd	[22, 43]
I113T	58.9 ± 12.6	Limbs	3.5 ± 2.8	nd	Y, pred	Y	NFT, ICAI, HC, NFCI	[22, 43, 50, 57, 238–240]
L126Z	58	nd	4	nd	Y	nd	LBHI	[58, 241]
G127X	50	Limbs	2.8	Y	Y	nd	nd	[242]

*nd* not described, *UMN* upper motor neuron signs, *LMN* lower motor neuron signs, *N* no, *Y* yes, *Pred* predominant, *LBHI* lewy-body-like hyaline inclusion, *NCI* neuronal cytoplasmic inclusion, *SLI* skein-like inclusion, *HC* hyaline conglomerate, *ICAI* intracytoplasmic argyrophilic inclusion (neurofilament accumulation), *NFCI* neurofilamentous conglomerate inclusion

**Table 2:** SOD1 animal models

Species	Mutation	Age at onset (weeks)	Survival (weeks)	Phenotype				References
				Paralysis	Cognitive symptoms	Neuropathological findings and particularities	Gliosis	
Mice	hSOD1^WT^	58–70	normal	N	nd	Mitochondria vacuolization and swelling,, spinocerebellar axonal degeneration, 20-30 % MN loss	Y	[69]
	hSOD1^WT^	36	52	Y	nd	SOD1 inclusions, vacuolization, MN loss, glial cell aggregates	Y	[71]
	A4V	>85	normal	N	N	nd	N	[70]
	A4V/SOD1^WT^	35	48	Y	nd	SOD1 inclusions, MN degeneration	Y	[70]
	G37R	15–17	25–29	Y	Learning deficit	MBV, LMN first affected, raised somatosensory thresholds	Y	[76, 243]
	H46R	20	24	Y	nd	LBHI, ubiquitin, SOD1 inclusions	Y	[244]
	H46R/H48Q	17–26	nd	Y	nd	HI, ubiquitin	nd	[245]
	H46R/H48Q/	35–52	nd	Y	nd	Fibrillary SOD1-ubiquitin inclusions	Y	[246]
	H63G/H120G							
	D83G^a^	15	70–84	N	nd	Sensory deficit, tremors, 20 % LMN and UMN loss	Y	[78]
	L84V	21–26	26–30	Y	nd	nd	nd	[247]
	G85R	35–43	37–45	Y	nd	Rapid progression, SOD1-ubiquitin inclusions in neurons and astrocytes	Y	[248]
	G85R/SOD1^WT^	16–21	23–30	Y	nd	SOD1 aggregates	Y	[249]
	G86R^b^	13–17	17	Y	nd	Rapid progression (5 days)	nd	[250]
	D90A	52	61	Y	nd	Distended bladder, SOD1 inclusions, MN loss	Y	[75]
	G93A	13–17	17–26	Y	Y	MN loss, SOD1 aggregates, NMJ loss before onset	Y	[61, 77]
	G93A/SOD1^WT^	20–23	25–30	Y	nd	Vacuoles, MN loss	Y	[69]
	Thy1.2-G93A^c^	54- >104	62- >104	N	nd	SOD1 aggregates	Y	[79]
	L126Z	28–44	47	Y	nd	Eosinophilic inclusion, MN loss, ubiquitin inclusions	Y	[70, 251]
	G127X	35	36	Y	nd	Rapid disease course, SOD1-ubiquitin inclusions	Y	[252]
Rats	H46R	20	24	Y	nd	MN loss, LBHI, SOD1-ubiquitin aggregates	Y	[253]
	G93A	16	17	Y	nd	MN loss, vacuoles, SOD1-ubiquitin inclusions	Y	[253, 254]
Dogs	T18S	7 years	21 months^d^	Y	nd	SOD1 aggregates No neuronal cell body loss, UMN and LMN signs, sensory impairment	Y	[86]
	E40K	>5 years	6 months–3 years^d^	Y	nd		Y	[85]
Zebrafish	A4V^e^	30 h	nd	N	nd	Motor axonopathy and abnormal branching	nd	[90]
	G37R^e^	30 h	nd	N	nd		nd	[90]
	G93A^e^	30 h	nd	N	nd		nd	[90]
	G93A	20–60	nd	N	nd	Increase time resting but no swim speed change, NMJ loss, 50 % MN loss	nd	[91]
	G93R^f^	12 months	18–27 months	partial	nd	NMJ defects, MN loss, swimming incapacity, vacuolated mitochondria	nd	[92]
Fruit flies	hSOD1^WT^	3	normal	N	nd	Loss of climbing, no MN loss, decrease synaptic transmission in giant fiber motor pathway	HSP70 stress response	[93]
	A4V	4	normal	N	nd			[93]
	G85R	2	normal	N	nd			[93]
	D83S	4	normal	N	nd	Mitochondrial pathology, decreased physical activity	nd	[255]
Nematodes	hSOD1^WT^	10 days	10–20 D	Y	nd	Reduction in thrash number	nd	[95]
	G85R	10 days	10–20 D	Y	nd	Forward movement defect, SOD1 inclusions	nd	[95]
	G93A	2 days	nd	Y	nd	SOD1 inclusions in MN, axons guidance defects	nd	[256, 257]
	C6S/C57S/	normal	normal	N	nd	No phenotype	nd	[95]
	C111S/C146S							
Pigs	G93A	12	normal	N	nd	MN loss at 8 months, Intra-nuclear SOD1-ubiquitin inclusions, running deficit, fibrillation potentials and positive sharp waves at EMG	Y	[99]

*Y* yes, *N* no, *nd* not described, *MBV* membrane-bounded vacuoles, *HI* hyaline inclusion, *MN* motor neuron, *LMN* lower motor neuron, *UMN* upper motor neuron, *LBHI* lewy-body-like hyaline inclusion, *EMG* electromyography, *NMJ* neuromuscular junctions

^a^Homozygous mouse *sod1* ^*D83G/D83G*^

^b^Mouse *Sod1* mutation

^c^Homozygous SOD1^G93A^ Thy1.2 promoter

^d^Disease progression

^e^mRNA SOD1 injection

^f^Zebrafish transgene

Mouse models have also been created to understand the role of SOD1 in specific central nervous system (CNS) cells. Neuron-specific high expression of SOD1^G93A^ under Thy1.2 promoter led to motor neuron degeneration and paralysis [79] but lower expression of SOD1^G37R^ under the NF-L promoter did not cause motor neuron deficit [80]. This inconstancy can probably be explained by level of hSOD1 expressed and the specific mutation used. A group also took advantage of the Cre recombinase to decrease expression of the SOD1^G37R^ mutant in astrocytes and they observed delayed microglial activation and slowed late disease progression [81]. Mice expressing the G86R mutant restricted to astrocytes did not develop motor deficit or microglial activation but showed increased Glial fibrillary acidic protein (GFAP) reactivity [82]. These results suggest that glial cells contribute to disease progression but are not sufficient to trigger motor neuron disease.

##### Other models

H46R and G93A are the only two human SOD1 mutations introduced in rat (Table 2). These rats developed ALS features similar to SOD1 mouse models. They develop UMN and LMN degeneration and similar neuropathological findings. SOD1^G93A^ rats also have motor neuron (MN) loss in trigeminal, facial and hypoglossal nuclei which correspond to features of bulbar ALS [83]. Rats are of particular interest for ALS research because of their size which facilitate intra-thecal or intra cerebro-ventricular injection for preclinical trials.

Canines can be affected by degenerative myelopathy (DM), a progressive neurodegenerative disorder with robust similarities to human ALS, making dogs the only mammals with naturally occurring non-human ALS [84]. DM is particularly similar to the UMN-dominant onset form of ALS. The onset of clinical signs generally occurred at 8 years of age and duration of disease rarely exceed 3 years because of elective euthanasia. T18S and E40K mutations in SOD1 were reported in dogs with DM [85, 86]. Similarities between both diseases include axonal degeneration, astrocytosis, fibrillation and positive sharp waves at electromyography evaluation, muscle atrophy, sensory impairment and SOD1 inclusions [84]. However, MN loss is not clearly established in DM as some authors reported MN loss in dogs with DM [87] whereas others did not [88].

Zebrafish have a simplified vertebrate nervous system which offers accessible motor neurons for *in vivo* study of ALS. They are particularly promising for fast screening of new potential therapeutic approaches as response to treatment can be rapidly observed. However, even if the zebrafish CNS shares similarities with human CNS, structural differences are important to consider when analyzing these models. Comparative neuroanatomy was recently reviewed in [89]. For example, the absence of corticospinal and rubrospinal tracts in zebrafish make it a poor model for UMN disorders. Zebrafish injected with mutant SOD1 mRNA display abnormal branching and reduced motor neuron axonal length, but did not become paralysed. In contrast to mice, zebrafish injected with the *SOD1* A4V mRNA exhibit the most prominent phenotype [90]. Transgenic G93A and G93R zebrafish exhibit MN and neuromuscular junction (NMJ) loss with deficits in swimming [91, 92].

*Drosophila melanogaster* is a useful tool for genetics research given its low maintenance cost as compared to rodents and other mammals. It also has shorter lifespan which allows for fast initial studies of diseases and treatment. However, the mammalian nervous system is much more complex and additional studies have to be realized in mammals before translating results directly from *Drosophila* to human. Human SOD1^WT^, SOD1^A4V^ and SOD1^G85R^ were expressed in *Drosophila* [93]. All of these transgenic flies exhibit climbing inability at two to four weeks of age, suggesting a motor deficit. A decrease in synaptic transmission was observed without MN loss. The authors also observed a stress response in glial cell through increased immunostaining of Hsp70.

*Caenorhabditis elegans* is a nematode which can be cultured in agar plates and reach a length of 1 mm [94]. *C. elegans* possess the advantage of being transparent thus allowing easy detection of fluorescent proteins. Their short lifespan permits the rapid screening of drugs and the extensive study of molecular pathways. However, the simplicity of their nervous system, as in *Drosophila* and zebrafish, do not allow the direct translation of discoveries and treatment to humans. Human SOD1^WT^, SOD1^G85R^, SOD1^G93A^ and SOD1 with mutations in four cysteine residues (C^4^S) were introduced into *C. elegans.* Motor neuron deficits were measured by the number of “thrashing” movements by the worm, which are periodical changes of direction when placed in physiological buffer. Overexpression of hSOD1^WT^ and SOD1^G85R^ caused motor deficit leading to paralysis within 10 to 20 days [95].

In last few years, pigs have been used for modeling disorders such as Parkinson disease [96], Alzheimer’s disease [97] and Huntington disease [98]. Their role in modeling disease is based on their anatomical, genetic and physiological resemblance to human. CNS anatomy is particularly closer to human than the rodents. Minipigs expressing hSOD1^G93A^ under the CMV promoter were produced [99]. From 3 months of age, the pigs develop hind limb defects resulting in running difficulty and this incapacity became more severe with age. The pigs also exhibit muscle atrophy and accumulation of SOD1 in motor neurons but did not die of the disease up to two years of age.

##### Biomarkers

Interest in biomarkers in ALS research has growth in the last decades. Unfortunately, no biomarkers are validated yet for the diagnosis of ALS. The discovery of biomarkers will help early diagnosis and potentiate therapeutic intervention in a context of personalized medicine. The optimized biomarker will be quantifiable, will be standardized, will have low variability and will be introducible within all centers. Volatile organic compounds (VOC), which are tools for biomarker investigation, have been analyzed in SOD1^G93A^ mice [100]. Mice, in early stage of the disease, exhibit a pattern of 12 different oxidative stress-related blood VOCs as compared to non-transgenic aged-matched mice. MicroRNAs (miRNAs) are also of particular interest in biomarker development as they are frequently altered in disorders, can be dosed in biological fluids and the pattern of variation in miRNA level is relatively specific. miRNA-206 has been identified in the blood and muscles of SOD1^G93A^ symptomatic mice [101]. miRNA-206 was also increased in blood of ALS patients, but without any discrimination of familial or sporadic form of disease. However, a recent report suggests that the miRNA pattern of expression in serum is mutation-independent. Consequently, stratified subgroups could increase the significance of miRNA as biomarkers. Indeed, patients with FUS and C9orf72 mutations have a similar serum miRNA profile compared to SOD1 patients, but profiles of miRNA was less similar among SOD1 patients [102]. Actually, among all ALS patients, a serum miRNA signature was observed decade before disease onset as compared to healthy controls. Imaging techniques are also potential biomarkers for ALS. SOD1^G93A^ mice exhibit a tissue vacuolization in T2 MRI studies. These alterations occurred before MN loss and matched histopathological change in post-mortem analysis [103]. Another group also observed vacuolization and gliosis in T2 MRI as soon as 60 days in SOD1^G93A^ [104].

##### Personalized medicine

Personalized medicine (PM) is a medical scheme that combines study of genetic, environmental and clinical diagnostic testing, including biomarkers, to individualize patient care. Patients are divided into stratified subgroups to improve their response to treatment [105]. This concept has emerged from the need to understand the variety of response to similar treatment in common illnesses. The application of personalized medicine in ALS first requires genetic screening among ALS patients in clinical trials and in overall neurology clinics and eventually, utilization of biomarkers for personalized diagnosis and treatment. Ideally, subgroups of specific mutations should be applied in clinical trials because of the high phenotypic variability of these mutations (Table 2). Unfortunately, recruitment difficulty is limiting this approach. As better drugs become available, it will be important to take into account the genetic profile status of the patient to determine if individuals with certain mutations would respond better to particular treatments. This has been successful in other diseases such as the use of Vemurafenib in melanoma patients specifically with a *BRAF* V600E mutation [106].

SOD1 animal models exhibits most clinical features found in humans with SOD1 mutations and this is particularly accurate for rodents and canine. This accuracy suggests that results in SOD1 rodents can potentially be applied to human with SOD1 mutations in a context of PM. However, the review of the literature suggest that SOD1 models are less accurate for modeling general ALS since they exhibit important disparities with sALS and other forms of fALS. SOD1 transgenic mice do not recapitulate the C-terminal and phosphorylated TDP-43 cytosolic inclusions observed in almost all familial and sporadic ALS cases [107, 108]. Likewise, Lewy body-like inclusions are a typical neuropathological finding of SOD1 patients and animal models but are not observed in sALS and SOD1-unrelated fALS [109].

Gene therapy is a promising avenue for personalized medicine in ALS. Recombinant adeno-associated viral (AAV)-mediated gene delivery is the most developed method for gene therapy. These vectors can target specific cells when directly injected in the CNS or by crossing the blood-brain barrier when injected systemically. AAVs expressing the insulin growth factor 1 (IGF-1) and glial cell line-delivered neurotrophic factor (GDNF) injected in muscle succeed to prolong life-span and delay disease in the SOD1^G93A^ mouse by retrograde action on MN [110]. Intra-thecal injection of AAV expressing a single-chain antibody against misfolded SOD1 also delayed disease onset and extended life-span up to 28 % in SOD1^G93A^ mice [111]. It is clearly established that SOD1 mutants develop a toxic gain of function. Consequently, silencing the *SOD1* gene by delivery of a lentiviral vector that expresses small interfering RNAs (siRNA) was tested in SOD1^G93A^. The authors described a delay in disease onset by more than 100 % and a life-span extends by 80 % [112]. A delay in onset and extension of life-span were also observed in SOD1^G93A^ rats injected with AAV9-SOD1-shRNA in the motor cortex [113] and in SOD1^G93A^ mice treated with intravenous injection of AAV9-SOD1-shRNA [114]. With the same idea, antisense oligonucleotide targeting the *SOD1* gene infused in the lateral ventricle of SOD1^G93A^ rats extends survival by 10 days and extends disease duration by 37 % [115]. Actually, a recent phase 1 clinical trial of intrathecal injection of antisense oligonucleotide was conducted in SOD1 familial ALS cases and no serious adverse effects were identified [116]. A phase 2 clinical trial is currently conducted but no results are yet available.

#### TAR-DNA-binding protein (TDP-43)

##### Specific disease characteristics in humans

TAR-DNA-binding protein 43 (TDP-43) is a multi-functional DNA/RNA binding protein normally found in the nucleus. It is known to play a role in RNA processing and transport and splicing regulation [117]. TDP-43 was found to be a major component of pathologic cytosolic inclusions in ALS and FTD [118, 119]. TDP-43 was subsequently found to be mutated in familial and sporadic ALS patients [120–123]. While TDP-43 mutations are rare (4 % of fALS), it is notably established that the TDP-43 protein is found in inclusions of most ALS cases. Over 40 mutations are currently identified but G298S, A315T, M337V, G348C, and A382T are the most frequent among patients [124].

It is currently clinically impossible to clearly discriminate TDP-43 patients from other sALS and fALS patients. It is also difficult based on the literature to establish clear phenotype-genotype correlation within each variant of TDP-43 mutation. Clinical characteristics of frequent mutations are outlined in Table 3. However, one publication succeeds to point out some specific clinical characteristic in TDP-43 mutated patients [124]. Age at onset can vary from 20 to 77 years old with a mean age at onset of 54 years old [125]. M337V and G348C have the earliest disease onset. Onset within TDP-43 patients is significantly earlier than sALS patients but no difference can be observed when compared to SOD1 patients, which also have earlier disease onset [124]. The upper limb is the predominant site at onset comprising 60.7 % of patients [124]. Bulbar onset seems more frequent in Asian patients (55 %) as compared to Caucasians (24.7 %) and in patients with a M337V mutation. Small cohort of patients with TDP-43 mutations was used and makes these results difficult to generalize.

**Table 3:** Clinical characteristics of human TDP-43 mutations reproduced in animal models

Mutation	Age at onset (years ± SD)	Site at onset	Survival (months ± SD)	Clinical manifestations				References
				UMN	LMN	Cognitive symptoms	Neuropathological findings	
G287S	62.3 ± 9.3	Bulbar, Spinal	76 ± 15.4	Y	Y	N		[121, 258, 259]
G290A	49.5 ± 2.1	Bulbar, Spinal	12^a^	Y	Y	N		[123]
G298S	50.7 ± 6.2	Bulbar, Spinal	27.0 ± 11.1	Y	Y	N	Bunina bodies, gliosis, neuronal loss anterior horn, NCI, GCI	[123, 124]
A315T	65.5 ± 13.6	Spinal	109.7 ± 32.3	Y	Y	N	Gliosis, spinal neuronal loss, NCI, neurofibrillary tangles, Aβ deposit	[120, 121, 124, 260]
Q331K^b^	72	Spinal	36	nd	nd	nd		[122]
M337V	47.7 ± 8.8	Bulbar, Spinal	105.0 ± 59.6	Y	Y	N	NCI, GCI, Bunina bodies	[122, 124, 258, 259, 261]
G348C	46.4 ± 10.7	Spinal	81.3 ± 37	Y	Y	Apathy, anxiety		[121, 124]
A382T	51.3 ± 8.6	Bulbar, Spinal	58.1 ± 44.4	Y	Y	Dementia		[121, 124, 126, 258]
N390D^c^	53	Spinal	35	Y	Y	N		[121]

*Y* yes, *N* no, *nd* not described, *NCI* neuronal cytosolic inclusion, *GCI* glial cytosolic inclusion

^a^Precise duration of disease was not mentioned

^b^Limb-onset ALS in a single case

^c^Limb-onset ALS in a single case

Fronto-temporal dementia is relatively rare within TARDBP ALS cases. A382T is the most described mutation in FTD-TDP-43 familial cases [126–129]. One group identified a Sardinian cohort of ALS with an A382T mutation with frequent dementia (30.8 %) [130]. They also identified that the A382T mutation is present in 21.7 % of their FTD cohort [126]. However, a similar frequency of TDP-43 mutations was not reported in other populations. The authors explained that this frequency is probably caused by the isolated population of Sardinia with its associated founder effects. Clinical features of FTD cases with an A382T mutation mainly include irritability, aggressiveness, poor flexibility, fixed ideas, change in eating behaviors, trouble in social behaviors and emotional flatness [126]. Brain imaging studies also suggested a pattern of defect found in A382T related FTD cases. MRIs showed unilateral or bilateral frontotemporal atrophy and frontotemporal hypoperfusion, observed in SPECT brain perfusion. In combination, neuroimaging findings showed prevalent involvement of temporal regions, which is rarely reported within other familial FTD cases [126].

Neuropathological findings in TDP-43 fALS patients tend to represent the majority of sALS and fALS cases. Regardless of the numerous publications on TDP-43, only a few reports are available with neuropathological findings. Indeed, most of our knowledge about TDP-43 neuropathological features comes from mouse models. In patients, neurons and glial cells are positive for TDP-43 cytosolic inclusions [131]. Often, these inclusions are also composed of ubiquitin and P62. Bunina bodies are described in most mutations except in A315T.

##### Mouse models

The discovery of TDP-43 as a major component of ALS inclusions had led to the generation of many mouse models trying to recreate this pathology (Table 4). In ALS patients, levels of the TDP-43 protein are elevated 1.5-2.5 fold in pathologic neurons [132, 133]. The first strategy for modeling ALS with TDP-43 transgenic mice was to overexpress hTDP-43^WT^ to similar level found in humans. Many available hTDP-43^WT^ models are based on high protein expression by the mouse prion (mPrp) or Thy1.2 promoters. The phenotype observed in these mice seems to be dependent on TDP-43 protein level and did not reproduce the age-related degeneration of ALS. Hemizygous mice expressing 3–4 fold levels of hTDP-43^WT^ did not recapitulate features of ALS [134]. These mice did not develop motor dysfunction and had only mild gliosis and diffuse ubiquitin staining. However, homozygous mouse expressing 2.5 fold increases in TDP-43 levels had rapid onset and low survival [135]. Mice died within approximately 2 months with associated motor deficits. Nevertheless, these mice did not recapitulate classical ALS neuropathological findings. Phospho-TDP-43 inclusions were rare and no motor neuron loss was observed. Mice expressing high level of hTDP-43^WT^ under Thy1.2 promoter (3.8–5.1 folds) also developed rapid progression of disease and had 30 % UMN and 25 % LMN loss with rare phospho-TDP-43 aggregates. Some reports have proposed that the fast disease progression without important loss of motor neurons could be caused by gastrointestinal complications before appearance of full neurodegeneration [136, 137].

**Table 4:** TDP-43 animal models

Species	Mutation	Promoter (fold expression)	Age at onset (weeks)	Survival (weeks)	Phenotype				References
					Paralysis	Cognitive symptoms	Neuropathological findings and particularities	Gliosis	
Mice	hTDP-43^WT^	mPrp (3–4)	None	Normal	N	nd	Diffuse ubiquitin staining, no NCI	mild	[262]
		**mPrp (2.5)** ^**a**^	**3**	**4–8**	**Y**	**nd**	**pTDP-43 NCI, cytosolic ubiquitination, axonal degeneration, no MN loss**	**Y**	[135]
		mThy1.2(3.8–5.1)	2–8	4–27	Y	nd	Rapid disease progression, rare pTDP-43 NCI, MN loss, phenotype correlates with protein level	Y	[263]
		mThy1.2(1.3–3.6)	Males: 2 Females: 13	nd	nd	nd	Mitochondrial aggregation, no TDP-43 NCI, decreased axon caliber, no MN loss	nd	[264]
		mCaMKII^c^ (0.4–1.7)	4	nd	nd	nd	Brain atrophy, Neuron loss, mosaic expression, rare pTDP-43 NCI	Y	[265]
		CAG	None	Normal	N	nd	No motor impairment, neuron loss in frontal cortex, no NCI	nd	[266]
		hEP (3)	42	Normal	N	Y	No TDP-43/ubiquitin NCI, motor dysfunction without paralysis	Y	[138]
	mTDP-43^WT^	mCaMKII(2)^a^	8	71	N	Y	Learning/memory deficit, TDP-43/ubiquitin positive NCI, progressive motor deficits	Y	[267]
	A315T\	mPrp (4)	4	10.7	Y	nd	Rare pTDP-43 NCI, no GCI, MA	Y	[134]
		**mPrp (3)**	**12–16**	**22 ± 2.7**	**Y**	**nd** ^**b**^	**Ubiquitin positive/TDP-43 negative NCI, UMN/LMN loss**	**Y**	[139]
		**hEP (3)**	**38**	**Normal**	**N**	**Y**	**TDP-43+/Ubiquitin + NCI at 10 months, peripherin inclusions, decrease axonal caliber, motor dysfunction without paralysis**	**Y**	[138]
		mEP (2.5)^d^	nd	nd	nd	N	TDP-43 NCI, 10 % MN loss	nd	[268]
	Q331K	mPrp (1.5)	12	Normal	N	nd	Decreased motor performance at 10 months, muscle fibrillations at EMG, No NCI	Y	[140]
	**WT/Q331K**	**mPrp (3.3)**	**3**	**8–10**	**Y**	**nd**	**TDP-43/ubiquitin/p62 NCI, 70 % MN loss in SC anterior horn**	**Y**	[141]
	M337V	mPrp (2.7)^a^	3	4	Y	nd	Tremors, pTDP-43 NCI, cytosolic ubiquitination, no MN loss, MA	Y	[269]
		mPrp (1.5)	40	Normal	N	nd	Decreased motor performance at 10 months, no NCI	nd	[140]
		Thy1.2 (1.7)^a^	<2	2,5	Y	nd	Ubiquitin/TDP-43 NCI, worse phenotype than TDP-43^WT^ mouse	Y	[270]
	**G348C**	**hEP (3)**	**36**	**Normal**	**N**	**Y**	**TDP-43+/Ubiquitin+ NCI at 10 months, peripherin inclusions, decreased axonal caliber, motor dysfunction without paralysis**	**Y**	[138]
Rats	hTDP-43^WT^	hEP (nd)	Normal	Normal	N	nd	Normal	nd	[142]
	M337V	hEP (nd)	2–3	1.5–4	Y	nd	Loss of MN in ventral horn	nd	[142]
	M337V	TRE (nd) PN day 4	3	5	Y	nd	Degeneration of ventral root, dorsal root and corticospinal tract, pTDP-43 staining, but no NCI	Y	[142]
		TRE-NFH(nd) PN day 60	10	nd	Y	nd	Paralysis within 3 weeks, no TDP-43 NCI, motor function restores with removal of TDP-43	Y	[143]
		TRE-GFAP(1.3) PN day 40	8.6	11.4	Y	nd	MN loss, MA,	nd	[271]
Fruit flies	hTDP-43^WT^	OK371-Gal4^e^ (nd)	10 days	nd	N	nd	TDP-43 inclusions, MN loss, larval motility deficit	nd	[272]
		D42-Gal4^e^(nd)	2–3	2.5–3.5	Y	nd	Progressive motor deficit leading to paralysis, no NCI	nd	[145]
		GAL4-UAS(nd)	nd	nd	Y	nd	Dose-dependent cytosolic TDP-43 and NCI, decreased larvae and adult movement, decreased NMJ	nd	[148]
	WT, Q331K	MN (nd)	nd	nd	nd	nd	Motor deficits, Q331K had worst phenotype	nd	[273]
	WT, F147L/F149L, G287S, A315T, G348C, A382T, ΔNLS	D42-Gal4^e^(nd)	10–20 days	20–40 days	Y	nd	Progressive loss of motor performance, worse phenotype seen in WT, MN loss	nd	[146]
	dTDP-43	D42^TS^-Gal4^f^(nd)	12–14 days	23 days	N	nd	Decreased thoracic number of neurons, locomotor defect, shorter lifespan	nd	[147]
Nematodes	TDP-1, hTDP-43WT	snb-1	larvae	nd	N	nd	No NCI, slow movement	nd	[149]
	hTDP-43WT, G290A, A315T, M337V	snb-1	nd	13–18.9 days	Y	nd	Lethargy, flattened sinusoidal waveform and reduced locomotion, worse phenotype with mutations, pTDP-43, ubiquitin, no NCI, nuclear aggregates	nd	[150]
	hTDP-43WT, A315T	unc-47	4–6 days	normal	Y	nd	GABAergic motor neurons expression, older paralysis in WT (20 days) as compared to A315T (12–13 days), MN loss, cytoplasmic TDP-43	nd	[207]
Zebrafish	hTDP-43WT, A315T, G348C, A382T	mRNAs	24 hpf	nd	Y	nd	Motor deficit, phenotype was mRNA concentration dependant, worse phenotype in mutant, decreased motor axons length	nd	[151]
	hTDP-43WT, A315T	mRNAs	28 hpt	nd	nd	nd	Reduced axonal length in A315T	nd	[152]

*Y* yes, *N* no, *nd* not described, *MA* muscle atrophy, *NCI* neuron cytoplasmic inclusion, *GCI* glial cytoplasmic inclusion, *hEP* human endogenous promoter, *mEP* mouse endogenous promoter, *MN* motor neuron, *PN* post-natal, *hpf* hours post-fertilization. Models in bold filled most of the quality criterias (see in main text)

^a^Homozygotes

^b^Ubiquitin inclusions were observed in cortex but not cognitive evaluation was realized

^c^TRE induction 28 days after birth

^d^Knock-in mice

^e^Motor neuron expression

^f^dTDP-43 expressed only at higher temperature

Our group took advantage of genomics fragments including the human endogenous TDP-43 promoter to moderately express hTDP-43^WT^, hTDP-43^A315T^ and hTDP-43^G348C^ in mouse [138]. These mice develop age-related progressive motor deficit in the rotarod test from 36–42 weeks of age and cognitive impairment suggestive of FTD. We also observed ubiquitin/TDP-43 nuclear and cytosolic inclusions in 10 month old hTDP-43^A315T^ and hTDP-43^G348C^ mice (Fig. 3). Nonetheless, these mice did not become paralysed and died at standard age. Interestingly, mPrp-hTDP-43^A315T^ mice develop disease and develop paralysis within few months, but no TDP-43 neuronal cytosolic inclusions (NCI) were observed [139]. As shown in Table 4, no clear correlation can be made between mutation and mouse phenotypes. Correlation can be observed though between the levels of protein expression and phenotypes. Low protein expression caused age-related motor dysfunction and TDP-43 cytosolic accumulation, but the mice did not get paralysed [138, 140]. On the other hand, high protein expression cause early onset and fast disease progression without clear motor neuron loss and TDP-43 NCI. More recently, double transgenic mice expressing hTDP-43^WT^ and hTDP-43^Q331K^ developed rapid progressive limb paralysis starting from 3 weeks of age and leading to death by 8–10 weeks [141]. TDP-43^WT^ and TDP-43^Q331K^ single transgenic mice do not develop motor dysfunction up to 24 months of age. These double transgenic mice also exhibited NCI positive for p62, ubiquitin and TDP-43, reproducing features of inclusions found in human cases. They also exhibited MN loss in the anterior horn of the spinal cord with association of muscular atrophy and NMJ loss. These mice recapitulated most ALS features in exclusion of age-related degeneration.

##### Other models

Few TDP-43 rat models have been generated. Mild expression of hTDP-43 with its endogenous promoter did not cause motor deficits in rats [142]. However, rats expressing the M337V mutation under endogenous TDP-43 promoter developed fast motor deficit following by paralysis and death within 10 to 29 days. To have a delayed phenotype and to successfully establish transgenic lines, the authors used a Tet-off system and activated TDP-43 expression 4 days after birth. These rats develop symptoms from 3 weeks of age and die at 5 weeks. TDP-43 was observed within the cytoplasm but no clear inclusions were detected. Interestingly, the rats exhibited degeneration of the ventral root, dorsal root and corticospinal tract. Specific neuronal expression of hTDP-43^M33V^ under the neurofilament heavy (NFH) promoter with a Tet-off system expressed at 60 days also caused rapid disease onset [143]. Rats became paralysed within 3–4 weeks after doxycycline induction of TDP-43 expression in neurons. Again, no TDP-43 neuronal cytoplasmic inclusions were detected but ubiquitin inclusions were detected in rats when hTDP-43^M337V^ was expressed in motor neurons.

Multiple *Drosophila melanogaster* models for TDP-43 have been generated to understand the cellular role of TDP-43. Many of them do not represent ALS because of their tissue expression and poor motor phenotype. They have been extensively described in a recent review on *Drosophila melanogaster* and ALS [144]. Interesting models of *Drosophila* were created with the D42-Gal4 system [145–147]. These flies expressed the TDP-43 transgene exclusively in motor neurons. hTDP-43^WT^ overexpression in *Drosophila* leads to fast paralysis and death in the larval stage [145]. Features of ALS were not clearly demonstrated since these flies died rapidly and no NCI or motor neuron loss was established. Later, hTDP-43^WT^ and hTDP-43^A315T^ overexpressing flies were found to have motor deficit in a climbing assay [146]. Interestingly, cytosolic aggregates of TDP-43 were observed in another model of hTDP-43^WT^*Drosophila* [148]. These flies also developed dose-dependent loss of NMJ with adult-onset motor disorder. They exhibit progressive loss of motor ability leading to complete paralysis at 30 days and control flies kept 20 % of motor aptitude at same age. Recently, thoracic motor neuron loss was observed in flies expressing temperature-dependant dTDP-43^WT^ [147].

Transgenic *Caenorhabditis elegans* models of TDP-43 are also based on protein overexpression. Two groups have expressed different forms of TDP-43 under control of the snb-1 promoter [149, 150]. They observed a motility defect and degeneration phenotype which was worse with mutant TDP-43. Some worms became paralysed and died within 3 weeks. Phosphorylated TDP-43 was detected but no TDP-43 NCI were noted. Moreover, injection of mutant TDP-43 mRNA in Zebrafish embryos caused motor neuron axonopathy [151, 152]. No phenotype was observed upon overexpression of WT-TDP-43 alone.

##### Biomarkers

Few TDP-43 biomarkers are available in literature and none of them are clearly validated for disease screening in human. ELISA quantification demonstrated an elevated TDP-43 protein level in plasma of ALS cases as compare to healthy subjects [153]. No differences were established between sporadic and familial cases. The same finding was observed in the cerebrospinal fluid (CSF) of ALS patients [154]. Interestingly, TDP-43 CSF level was higher within the first 10 months of disease onset. TDP-43 CSF level was significantly higher than the level measured in other neurologic disorder such as Parkinson’s disease, multiple sclerosis, Guillain-Barré syndrome and progressive supranuclear palsy [155]. A limit dose of 27.9 ng/ml of TDP-43 protein in CSF had a sensitivity of 59.3 % and a specificity of 96.0 %. These results suggest that TDP-43 CSF level could be an interesting tool for ALS diagnosis. Recently, tissue-engineered skins derived from ALS patients demonstrate that skin could possibly be used as biomarker as well. TDP-43 aggregation was observed in sALS-derived skin and in not yet symptomatic patients carrying GGGGCC DNA repeats [156].

##### Personalized medicine

According to our review of the literature, most TDP-43 models exhibit only a part of all important ALS features. We used five major criteria to point out the mice models which are more representative of human disease. Mice should have TDP-43 cytosolic inclusions, motor neuron loss, age-related onset of disease, paralysis which cause shorter lifespan, and gliosis. In most models, the rapid onset does not correlate with development of ALS in older patients and thus is difficult to use as a neurodegenerative model. Generally, mice with late onset do not get paralysed and miss one the major aspect of ALS pathogenesis. We have highlighted models which filled most of the criteria (**Bold** in Table 4). We consider that these models should be preferred for drug testing.

Promising approaches for the treatment of TDP-43 cases have been generated in the last few years. We have highlight therapies which can be applied to specific treatment of TDP-43 cases in a context of personalized medicine. Induced pluripotent stem cells (iPSCs) derived from TDP-43^M337V^ patients have been used for gene therapy testing [157]. A reduction of 30 % of cytosolic TDP-43 and 45 % of nuclear TDP-43 level was observed in M337V-iPSCs transfected with a siRNA specifically targeting M337V allele. This approach has to be tested in animal models but seems to be a potential treatment of TDP-43 fALS cases. IPSCs will certainly help to develop therapies in the next years. Specific mutations in patients could be targeted by siRNAs. It is well accepted that TDP-43 mutations disturb cellular RNA metabolism. One of the surveillance mechanisms in cells is composed of upframeshift protein 1 (UPF1) which destroys mRNAs with premature codon. AAV-expression of UPF1 in TDP-43 mice ameliorates motor phenotype and blocked paralysis of forelimbs [158]. These results suggest that gene therapy could also be used for overexpression of surveillance mechanisms instead of directly targeting the TDP-43 protein.

#### C9ORF72

##### Specific disease characteristics in humans

A hexanucleotide (GGGGCC) repeat in a non-coding region of C9orf72 was first described in 2011 in ALS, FTD and ALS-FTD familial cases [159, 160]. C9orf72 frequency varies geographically but seems to be present in up to 35–45 % of fALS, making it the most common genetic cause. The normal number of GGGGCC repeats is variable within healthy persons; approximately 90 % have fewer than 10 repeats. Conversely, the number of repeats can reach hundreds to thousands in ALS patients. The role of these repeats in ALS-FTD physiopathology remains unclear. However, recent semi-automated quantification of expansion number exposed a link between the number of G_4_C_2_ repeats and clinical characteristics [161]. FTD patients have shorter disease duration with a higher number of repeats, but no correlation was observed in ALS patients.

C9orf72-related ALS cases have several distinct characteristics. Nevertheless, like other genes, a wide range of clinical phenotype can be observed in patients carrying C9orf72 expansions. Age at onset of C9orf72 (C9ALS) fALS patients vary between studies [162–164]. There is good evidence that bulbar onset is more frequent in C9ALS patients than non-C9orf72 patients [162, 164, 165]. Also, there is certainly a higher prevalence of FTD in C9ALS cases. Co-morbid dementia was observed in 50 % of C9orf72 patients and only in 12 % of ALS cases without the expansion [164]. C9orf72-related FTD is mainly characterized by a higher frequency of psychotic symptoms and irrational behaviour as compared to other causes of FTD [166]. Interestingly, hypokinesia/bradykinesia, rigidity and sometimes, tremor, suggestive of parkinsonism, have been described in C9ALS patients [167]. Members of this family were either touched by ALS, FTD and/or progressive supranuclear palsy (PSP). Generally, parkinsonism symptoms seen in these patients are unresponsive to levodopa treatment. C9orf72 expansion was rare in the diagnosis of Parkinson’s Disease (PD) [168, 169].

Particular features can be seen in imaging studies of C9orf72-related ALS/FTD and are dependent of the presence or absence of FTD symptoms. PET imaging identified hypometabolism in anterior and posterior cingulate, insula, caudate and thalamus, and hypermetabolism in the midbrain, bilateral occipital cortex, globus pallidus and left inferior temporal cortex as compared to sALS patients [170]. Voxel-based morphometry revealed atrophy in right frontal gyrus, in left anterior cingulate gyrus and in right precentral gyrus of C9ALS patients [164].

TDP-43 pathology is largely observed in the motor system of C9ALS patients [171]. There is also a large amount of P62 positive and TDP-43 negative inclusions in the pyramidal cells, hippocampus and cerebellum of C9ALS patients [172]. A recent review of the literature has help to understand the neuropathological findings associated with G_4_C_2_ repeat cases [173]. It is important to note that while the G_4_C_2_ repeat occurs within intron 1 of the C9orf72 gene, the repeat expansion has been shown to nonetheless be translated in both sense and antisense orientations to generate proteins with dipeptide repeats that then are found in intracellular inclusions. The prevalence of TDP-43, p62 and dipeptide protein repeat (DPR) inclusions was assessed specifically in the CNS. In the spinal cord, TDP-43 NCI was observed in 84.1 %, P62 NCI in 70.6 % and DPR inclusions in 49.8 % of patients. P62 and DPR were the major component of NCI in the cerebellum (93.8 and 90.9 % respectively) and TDP-43 was found in only 3.9 % of cerebellums. P62 and DPR were more prevalent than TDP-43 in the hippocampus but TDP-43 was more prevalent in the substantia nigra.

##### Mouse models

The first mice carrying GGGGCC repeats expansions were recently generated [174] (Table 5). These mice carried 80 G_4_C_2_ repeats controlled by TRE promoter. After doxycycline induction, the mice developed ubiquitin-positive inclusion but no DPR were observed. The authors suggest that this model can further be used for the study of toxicity induced by RNA. However, no behavioral analysis was conducted on these mice. Knockout of *C9orf72* in mice did not result in any motor neuron degeneration or reduced survival, which suggests that loss-of-function is not sufficient to cause ALS [175]. More recently, an AAV vector expressing either 2 or 66 repeats of G4C2 were injected into CNS of postnatal day 0 mice [176]. RNA nuclear foci were observed in mice carrying 66 repeats but not in control mice carrying only 2 repeats. Also, rare pTDP-43 aggregates were observed in the nucleus and cytosol of cortex and hippocampus regions. These aggregates were not positive for poly(GA) but 75 % of cells containing TDP-43 NCI were positive for poly(GA) inclusions. (G_4_C_2_)_66_ mice exhibit anxiety behavior in an open field test and motor impairment in the second day of rotarod testing at 6 months of age as compared to control mice.

**Table 5:** C9ORF72 animal models

Species	Number of repeats	Promoter	Age at onset (weeks)	Survival (weeks)	Phenotype				References
					Paralysis	Cognitive symptoms	Neuropathological findings and particularities	Gliosis	
Mice	80	TRE	none	normal	N	N	ubiquitin-positive inclusion, no DPR, no TDP-43 inclusion	nd	[174]
	66^a^	nd	24	nd	nd	Y	Nuclear RNA foci, phosphoTDP-43 inclusions, cytosolic and nuclear DPR, Anxiety and social abnormalities, motor impairment	Y	[176]
	100–1000	BAC^b^	none	normal	N	N	RNA foci, DPR, no NCI	N	[178]
	500	BAC^b^	none	normal	N	N	RNA foci, DPR, no NCI	N	[177]
	450	BAC^b^	52	normal	N	Y	RNA foci, DPR, age-dependant protein accumulation, no motor deficits or MN loss, age dependant cognitive deficit, no TDP-43 mislocalization	N	[179]
	500	BAC^b^	16	20–40^c^	Y	Y	NMJ loss, reduced axonal size, MN loss, RNA foci, DPR, TDP-43 NCI	Y	[180]
Fruit flies	36–103	elav-GS	nd	30 days	nd	nd	RNA foci, DPR, toxicity was attributed to DPR	nd	[182]
	160	actin5C-Gal4	none	normal	N	nd	RNA foci, DPR, toxicity was attributed to DPR	nd	[181]
	30	Ok371-Gal4	4	nd	nd	nd	Decreased locomotor activity	nd	[183]
	58	Ok371-Gal4	nd	nd	nd	nd	Decreased locomotor activity, NMJ loss, DPR	nd	[184]
Nematodes^d^	n/a	alfa-1	2	nd	Y	nd	MN loss, paralysis in 60 % of worms	nd	[185]
Zebrafish^e^	n/a	n/a	nd	nd	nd	nd	MN axons shortening, reduced swimming	nd	[186]

*Y* yes, *N* no, nd not described, *n/a* not applicable, *BAC* bacterial artificial chromosome, *DPR* dipeptide repeat, *NCI* neuron cytoplasmic inclusion, *MN* motor neuron, *NMJ* neuromuscular junction

^a^Intracerebroventricular injection of AAV2/9 containing 66 G_4_C_2_ repeat lacking ATG start codon at post-natal day 0

^b^BAC include sequence of the human *C9ORF72* exons with promoter

^c^35 % of female mice died from 20–40 weeks, most of other mice develop cognitive symptoms at older age

^d^ *C. elegans* orthologue *alfa-1(ok3062)* mutation

^e^Knockdown of zebrafish C9orf72 orthologue (C13H9orf72)

Recently, two different groups generated mice carrying a bacterial artificial chromosome (BAC) with 100 to 1000 GGGGCC repeats [177, 178]. These mice did not exhibit any behavioral or motor phenotypes. However, they developed sense/antisense intranuclear RNA foci and DPR in both neurons and glial cells. Both groups suggest that this number of repeat is sufficient to cause RNA accumulation but not to cause cellular dysfunction leading to motor disease in mice. ALS patients with only a few hundred copies have been reported. Thereby, this discrepancy between mice and human has to be clarified. Very recently, two other groups generated transgenic mice carrying BAC with approximately 450 and 500 GGGGCC repeats [179, 180]. Jiang et al. have generated a mouse model that exhibits cognitive deficits from 12 months of age in working memory test and anxiety evaluation [179]. The mice also exhibit age-related RNA foci and DPR cytosolic accumulation. Liu et al. have generated and well characterized a mice model which developed many features of C9ALS [180]. These mice developed muscle denervation, MN loss, anxiety-like behavior, degeneration in hippocampus, paralysis and decreased survival. RNA accumulation was age-related and the authors also found TDP-43 inclusions in degenerating brain.

##### Other models

Whether nuclear RNA foci or DPR, or both, are toxic for cells remain an important question and this is currently under investigation by many groups. As previously described, *Drosophila melanogaster* is a superior tool for understanding pathological mechanisms than mimicking disease phenotype. Two recent publications suggested that DPR are more toxic to cells than nuclear RNA foci by generation of transgenic drosophila [181, 182]. In both studies, drosophila containing DPR had decreased survival but no phenotype was observed in flies with expression of RNA-only repeats. However, motor impairment was not described. 30 GGGGCC repeats were expressed under the motor neuron specific promoter Ok371-GAL4 in *D. melanogaster* [183]. With *Drosophila* activity monitoring (DAM) system, the authors observed a significant motor impairment at 28 days after eclosion. Neuronal death was observed in cell culture and in the eye but was not confirmed in motor neurons of flies. Another drosophila model was generated with 58 repeats of G_4_C_2_ in motor neurons using the Ok371-GAL4 promoter [184]. These flies developed impaired locomotor activity in larvae, a decrease in bouton number of NMJ and a low muscle area.

Deletion of the C9orf72 orthologue, *alfa-1(ok3062)*, in *C. elegans,* caused a motor phenotype [185]. The worms developed an age-related motility defect reaching paralysis in 60 % of worms at 12 days of adulthood. The authors also crossed *alfa-1(ok3062)* with their FUS^S57Δ^ and TDP-43^A315T^ model and observed that the motor phenotype was worse in the double transgenic *alfa-1(ok3062)*;TDP-43^A315T^*C. elegans* as compared to simple TDP-43 mutant worms. However, no synergistic effect was observed in *alfa-1(ok3062)*; FUS^S57Δ^ worms. The loss of function theory was also studied in zebrafish. Knockdown of the zebrafish C9orf72 orthologue was established using antisense morpholino oligonucleotides [186]. Fish developed motor neuron axon abnormalities, swimming impairment in reaction to touch and also reduced spontaneous swimming 48 h post-fertilization. No information was given on survival of zebrafish.

##### Biomarkers

Multiple biomarkers have been studied as diagnostic tool or disease progression marker in C9orf72 pathology despite the relatively recent discovery of its implication in ALS. One of the most characterized cellular findings within C9orf72 patients is the presence of nuclear RNA foci. RNA foci are found in many cell types such as skin biopsy-derived fibroblasts, leukocytes and lymphoblasts and can possibly be utilized as a disease progression marker [187]. Also, a recent report suggests that the level of 5’ methylation of the G_4_C_2_ repeat can inversely correlate with disease duration in 34 C9orf72 patients [188]. This epigenetic modification can be found in blood, spinal cord and frontal cortex. Another potential biomarker tool is the level of poly-GP proteins in a patient’s CSF [189]. When compared to healthy subjects and ALS patients without C9orf72 repeat expansion, C9-ALS patients have significantly increased levels of poly-GP peptide in their CSF. Further studies have to be performed to clarify peptide levels in each disease progression stage.

##### Personalized medicine

It is premature to establish which C9orf72 model is better for mimicking human disease. Animal models of C9ALS repeat expansion have to be optimized and extensively characterized. However, based on clinical presentation, some specific characteristics are central features of C9 cases and should be found in animals. This includes RNA foci, DPR and TDP-43 positive aggregates, age-related disease and reduced survival. Cognitive symptoms are also commonly found in C9ALS patients. Most generated mice develop cellular features of C9ALS patients such as RNA foci and DPR [176–178]. Recently, two groups have generated promising models which exhibits most of C9ALS features and might be exploited for drug testing [179, 180].

Although many therapeutic targets are conceivable, only a few have been currently tested. First, targeting RNA with antisense oligonucleotide (ASO) therapeutics should be a potential avenue because of RNA toxicity in C9ALS models. ASO were tested in fibroblasts and iPSCs derived from C9ALS ALS patients and succeed to decrease RNA foci without reducing overall RNA levels [190, 191]. ASO managed to reduce glutamate toxicity and increased survival of iPSCs by 30 % [190]. Tolerability of C9orf72 reduction in mouse was then tested and no pathological effects were observed on motor ability, strength and anxiety up to 17 weeks after ASO treatment by intracerebroventricular injection [191]. More recently, a single-dose ASO injection which targets repeat-containing RNAs caused a reduction in RNA foci and DPR in mice expressing BAC C9ORF72 RNA with 450 repeats [179]. Reduction in anxiety and cognitive function were also observed at 9 months and a positive effect on behavioral was sustained 6 months after injection. These results suggest a promising treatment possibility for *C9orf72* patients.

#### Fused in sarcoma (FUS)

##### Specific disease characteristics in humans

Genetic screenings have identified mutations in the gene encoding fused in sarcoma (*FUS*) [192–194]. Similarly to TDP-43, the main function of FUS is linked to RNA metabolism. More than 50 mutations have been identified and mutations in FUS represent around 4 % of fALS and 1 % of sALS. Unfortunately, there are only a few clinical descriptions of FUS pathology and phenotypic correlation is difficult to establish. Many reports suggest that juvenile onset is more frequent in FUS cases. A German study reports a series of families with age at onset ranging from 21 to 76 years old and with many cases before the age of 40 [195]. They also suggest that truncating mutations have a more severe disease course than missense mutations. LMN signs appear to be the dominant features in FUS cases. Cognitive symptoms are particularly rare with only few cases reported in the literature and bulbar/spinal onset are both frequent with a mutation in *FUS* [196].

Neuropathological findings in FUS-related disease include basophilic inclusions which are round or oval often similar in size to the nucleus. These inclusions are mainly found in the anterior horn of the spinal cord and sometimes in the motor cortex [197]. These inclusions are commonly found in juvenile ALS with mutations in FUS. NCI are also present in pathological analysis and are similar to those observed in TDP-43 pathology. They are often positive for p62 but negative for TDP-43 [32].

##### Mouse models

FUS KO mice were reported to investigate the effect of FUS deletion. Mice either died in the first 24 h of life or had important deficits including sterility and chromosomal instability [198]. Another group suggested that knock-out FUS mice were developing a neuropsychiatric disorder that did not correspond to ALS [199]. Overexpression of hFUS^WT^ under the mouse prion promoter leads to an aggressive phenotype in homozygous mice [200] (Table 6). These mice had approximately 1.9 times the level of FUS expression. They develop motor impairment leading to paralysis from 8 weeks of age and were euthanized when 10–13 weeks old. They exhibit increased cytoplasmic FUS without ubiquitinated-FUS inclusions, MN loss in the anterior horn of the lumbar spinal cord, impaired NMJ and gliosis. Mice expressing FUS^WT^ or FUS^R521G^ driven by the ubiquitously expressed enhancer-chicken β-actin hybrid (CAG) promoter were also generated [201]. The mice exhibited early mortality before post-natal day 30 without any FUS inclusions or degeneration of lateral columns and ventral horns. However, gliosis and motor impairment in rotarod tests were observed.

**Table 6:** FUS animal models

Species	Mutation	Promoter	Age at onset (days)	Survival (days)	Phenotype				References
					Paralysis	Cognitive symptoms	Neuropathological findings and particularities	Gliosis	
Mice	KO	n/a	nd	nd	nd	nd	24 h death, chromosomal abnormality, sterility	nd	[198]
	KO	n/a	nd	nd	nd	Y	No motor phenotype, hyperactivity behavior	nd	[199]
	hFUS^WT^	mPrp	4 weeks	10–13 weeks	Y	N	Tremors, weight loss, deficit in rotarod, increase cytoplasmic FUS signal, spinal MN loss, NMJ loss	Y	[200]
	hFUS^WT^, R521G	CAG	10	30	N^a^	Y	More lethality in FUS^WT^, no MN loss in lateral column, MA, NMJ loss, reduced social interaction and motor performance	Y	[201]
Rats	hFUS^WT^, R521C^b^	TRE	27–48	33–55	Y	Y	Few MN loss, MA, NMJ loss, ubiquitin inclusions, no FUS inclusions	Y	[202]
Fruit flies	hFUS^WT^, R524S, P525L	OK371-Gal4	nd	nd	nd	nd	Large MN, decreased locomotor function, NMJ loss	nd	[203]
	hFUS^WT^, R518K, R521C, R521H	OK371-Gal4/elav-GS	10	~17	nd	nd	Decreased locomotor function, no NMJ loss, more cytosolic FUS in mutant,	nd	[204]
Nematodes	hFUS^WT^, R514G, R521G, R522G, P525L, FUS513, FUS501	P*rgef-1*	3	8.1–9.7	Y	nd	NCI of mutant FUS, worst phenotype in R522G, P525L, FUS513 and FUS501	nd	[206]
	S57Δ	*unc*-47	12–13	normal	Y	nd	MN loss, FUS insoluble aggregates	nd	[207]
Zebrafish	R521C, R521H, S57Δ	mRNA	48 hpf	nd	nd	nd	Reduced swimming at TEER, NMJ loss	nd	[208, 209]

*hFUS* ^*WT*^ rats develop cognitive symptom at one year of age, *Y* yes, *N* no, *nd* not described, *n/a* not applicable, *MN* motor neuron, *NMJ* neuromuscular junction, *MA* muscle atrophy, *NCI* neuronal cytosolic inclusion, *TEER* touch-evoked escape response, *hpf* hours post-fertilization

^a^Reduced grip strength and hindlimb clasping

^b^Only hFUS^R521C^ rats develop paralysis and features of ALS

##### Other models

An encouraging hFUS^R521C^ rat model was generated using a tetracycline-inducible system [202]. After doxycycline withdrawal, the mutant FUS protein was expressed in offspring. The rats developed many features of ALS pathology. Degenerating axons were observed in the spinal cord ventral roots, dorsal roots, corticospinal tracts and frontal cortex of hFUS^R521C^ rats but not in hFUS^WT^ rats. Muscle atrophy was observed and electromyography shown fibrillation potential which is suggestive of muscular atrophy. FUS-negative ubiquitin aggregates were detected in transgenic hFUS^R521C^ rats. Glial activation was also detected in the brain and spinal cord. Nevertheless, the rats developed fast paralysis leading to death within 30 to 70 days of age.

Several *Drosophila melanogaster* models were generated for investigating the pathological role of FUS. First, the Gal4-UAS system was used for expression of WT, R524S or P525L mutations in motor neurons [203]. Morphological changes were observed in the cell body and at NMJ leading to functional deficits of MN in transgenic flies. The authors observed significant locomotive impairment in larval movement in flies expressing either of three FUS variants. Conditional expression of FUS variants with the elav-GeneSwitch system demonstrated a shorter lifespan of transgenic flies [204]. The authors observed a faster rate of death and presence of cytosolic accumulation of FUS protein in flies expressing mutated FUS as compared to flies expressing WT-FUS. Interestingly, the level of non-aggregated but insoluble FUS protein positively correlated with the level of neurodegeneration in transgenic *Drosophila* [205].

*Caenorhabditis elegans* was also used for modeling FUS pathology and interesting results were obtained from this model. A group expressed different forms of FUS including WT, R514G, R521G, R522G, P525L and two truncated FUS [206]. Only R522G, P525L and the truncated forms of FUS caused aggregates and only these transgenic worms exhibited a motor phenotype. Motor impairment started at 3 days of age and *C. elegans* became paralysed at 8 days of age. Survival was reduced by 12.8 days in worms expressing R522G, P525L or truncated FUS as compared to non-transgenic *C. elegans*. These results were partially confirmed by expression of either WT or S57Δ FUS in GABAergic neurons [207]. Motor phenotypes were observed only in FUS^S57Δ^ worms which expressed FUS cytosolic inclusions.

Zebrafish harboring mutations in FUS gene were also generated. FUS^R521H^ but not FUS^R521C^ or FUS^S57Δ^ fishes exhibited motor impairment when compared to FUS^WT^ [208]. High-speed video analyses of touch-evoked locomotor activity revealed shorter swim duration, swim distance and swim velocities in fish expressing FUS^R521H^ as compared to wild type fish [209]. NMJ synaptic transmission was also reduced.

##### Personalized medicine

Our review of the literature has pointed out rat and mouse models with many phenotypic and neuropathological features of ALS [200, 202]. However, they do not possess age-related characteristic of ALS. We consider that these models could be adequate models for drug testing relative to FUS pathology. There is sparse literature which can be applied to a personalized medicine approach in FUS cases. One group has tried to identify biomarkers in skin derived fibroblasts from sporadic ALS patients. Unfortunately, the study failed to identify differences in FUS pattern of expression in skin between healthy control and ALS cases [210]. Like previously described, mi-RNA are potential biomarkers in ALS. FUS is known to be implicated in metabolism of a subset of micro-RNAs such as miR-9, miR-125b and miR-132 [211]. Their value as biomarkers remains to be tested. Methylation is an important process in nuclear-cytoplasmic shuttling of FUS. One group tried to reduce FUS cytosolic accumulation by reducing their methylation with shRNA targeting the FUS methylation enzyme [212]. Reduction of FUS cytoplasmic inclusions was noted.

#### Other genes

Multiple other genes were identified in familial ALS cases including Ubiquilin-2 and Optineurin (Fig. 1). These mutations remain rare and little data are available for the clinical presentation of these forms. Thus, more work has to be done before a personalized medicine approach in these patients can be comprehensively reviewed.

##### Ubiquilin-2 (UBQLN2)

Ubiquilin-2 plays a central role in the ubiquitin proteasome system (UPS). Mutations in *UBQLN2* have been linked to ALS and FTD [213] though screening in different populations has revealed that mutations in *UBQLN2* are generally rare within ALS cases [214, 215]. However, the UBQLN2 protein is found in cytosolic inclusions of both familial and sporadic ALS and appears to have an important role in pathological processes such as aggregate formation and proteasome impairment [213, 216]. No clear genotype-phenotype correlation can be established in patients. FTD appears to be frequent in ALS caused by mutation in *UBQLN2*. Both males and females can be affected despite the X-linked transmission [217]. Some studies have suggested early age at onset in familial UBQLN2 ALS and site at onset was described in lower limbs, upper limbs and bulbar regions [218]. A specific pattern of UBQLN2 staining has been observed in C9orf72 patients and the authors proposed that this staining could be used as biomarker for identification of C9orf72 cases [219].

Mice carrying a hUBQLN2^P497H^ mutation under control of the UBQLN2 endogenous promoter were recently generated [220]. These mice develop UBQLN2/ubiquitin/p62 positive inclusions in the brain, dendritic spinopathy and cognitive deficits at 11–13 months of age which suggest features of FTD. However, the mice do not develop motor impairment or motor neuron loss. Conversely, AAV expression of three different UBQLN2 variants by intracerebroventricular injection in mice caused a phenotype at 3–4 months of age [221]. Transgenic UBQLN2^P497H^ or knock-out rats were also created [222]. While cognitive and neuronal loss was observed in UBQLN2^P497H^ rats, no phenotype developed in knock-out rats. Astrocytes and microglial activation was observed but no information was given about motor function.

##### Optineurin (OPTN)

The Optineurin (OPTN) protein is mainly implicated in the autophagy processes. A mutation in the *OPTN* gene was first linked to ALS in a consanguineous Japanese family [223]. A neuropathological study suggested that OPTN was present in skein-like inclusions and round hyaline inclusions in the spinal cord of sALS patients [224]. However, another study mentioned that OPTN inclusions are rare and restricted to a minority of sALS cases [225]. Clinical characteristics were investigated in patients carrying Q398X and E478G mutations [226, 227]. These patients exhibit slowly progressive motor dysfunction, unusual finger malformations and personality changes. Observed NCI were positive for TDP-43, p62 and ubiquitin but negative for OPTN. OPTN inclusions were described in a patient with both C9orf72 hexanucleotide repeats and an OPTN mutation [228]. Both spinal and bulbar onsets are described but no strong conclusions can be made about duration of the disease and age at onset. Generation of a transgenic mouse with mutation in OPTN gene failed to demonstrate any motor phenotype [229]. Similarly, loss of OPTN in Zebrafish results in cell death but no motor phenotypes were noted [230].

## Conclusion

ALS is a fatal disease with large genetic and phenotypic heterogeneity which leads to a variety of responses to similar treatment regimens. There is currently a strong need for treatment discovery to help patient care. For that purpose, animal models which exhibit human disease characteristics have to be optimized. Most of current identified genetic mutations have corresponding animal models. We hope that this review will increase the awareness on qualities and weakness of these models and will eventually help researchers to take advantage of the best model available.

**Fig. 4:**
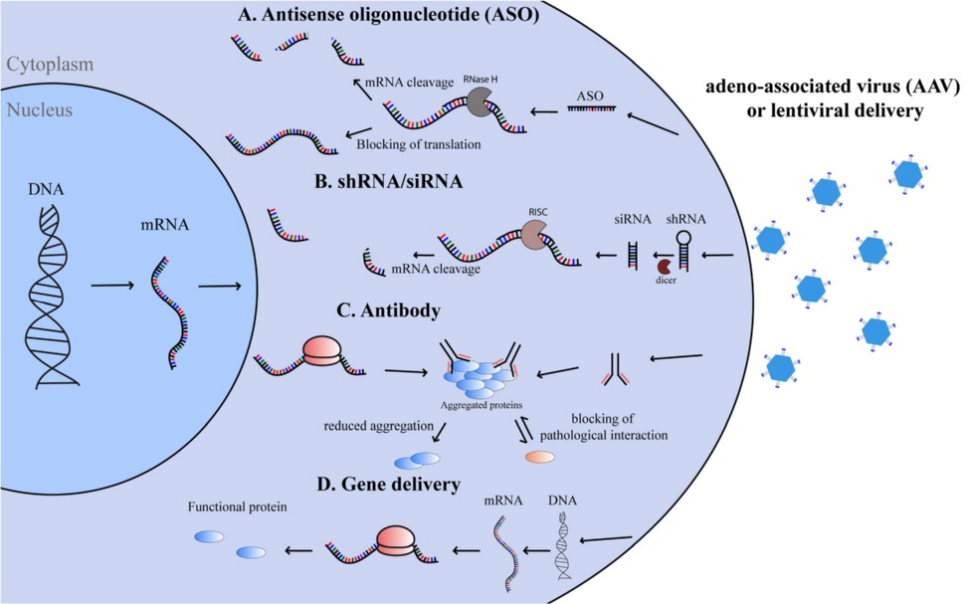
Gene therapy mechanism of action. Schematic representation of possible gene therapy approaches in ALS treatment. All of these approaches can be effective by intra-thecal, intracerebroventricular or peripheral injection of AAV or lentivirus targeting motor neurons or glial cells. **a** Antisense olinucleotide (ASO) are short synthetic oligonucleotides (15-25 nucleotides) which bind to targeted mRNA. ASO reduces the expression of a specific protein by two main mechanisms. ASO induces the mRNA degradation by endogenous RNase H or blocks the mRNA translation. This is a potential therapeutic avenue in ALS by reducing the protein level of TDP-43, SOD1 of FUS protein level or by targeting of C9orf72 RNA foci. **b** SiRNAs are double-stranded RNAs which operated through RNA interference pathway. After strand unwinding, one siRNA strand binds argonaute proteins as part of the RNA-induced silencing complex (RISC) and is recruited to a target mRNA which is then cleaved. **c** Antibodies are another potential therapeutics avenue in ALS [111]. Antibodies can target misfolded proteins and reduce the amount of toxic aggregates. It is suggested that they can reduces the disease propagation between cells. They can also be exploited to block the pathological interaction between proteins by binding to the specific interaction sites. **d** Gene delivery is another potential therapeutic avenue for loss-of-function mutations. Virus can provide a functional replacement of a missing gene by mRNA or cDNA delivery. This approach was particularly tested in spinal muscular atrophy and revealed great outcomes but is not yet extensively tested in ALS [231]

Personalized medicine approaches allow physicians to group together patients with similar characteristics. This could be performed with the use of biomarkers and over time with the same mutated gene. We have reviewed specific treatments which could be applied to sub-groups of patients with ALS. We consider that gene therapy has great potential for personalized medicine approaches, either by antisense oligonucleotide, small interference RNA or any other method such as antibodies targeting pathological proteins (Fig. 4). These techniques have already been tested and appear to be effective in SOD1, TDP-43, C9ORF72 and FUS animal models [111–115, 157, 179, 212]. We are optimistic that the use of gene therapy will growth in clinical trials in the next few years. Promising technologies for delivering genes have been suggested and revealed many procedures for safely targeting central nervous system. Lentiviral or AAV injections or peripherally injected exosomes which specifically target neurons are within these auspicious avenues.

At the moment, it is obvious that patients with ALS would welcome the possibility of any general treatment before having to be excluded based on their genetic status or some other criteria. However, we consider that the achievement of successful clinical trials for any treatment could be increased with sub-groups of patients established on genetic screening and biomarkers without excluding any patients. Clearly, more work has to be performed before appropriate clinical use of biomarkers but we expect that research will be improved in the next years.

## Abbreviations

AAV, adeno-associated viral; ALS, amyotrophic lateral sclerosis; ASO, antisense oligonucleotide; BAC, bacterial artificial chromosome; CNS, central nervous system; CSF, cerebrospinal fluid; DM, degenerative myelopathy; DPR, dipeptide repeat protein; DTI, diffusion tensor imaging; EEC, El escorial criteria; fALS, familial amyotrophic lateral sclerosis; FTD, frontotemporal dementia; FUS, fused in sarcoma; GDNF, glial cell line-delivered neurotrophic factor; GFAP, glial fibrillary acidic protein; IGF-1, insulin growth factor 1; iPSCs, induced pluripotent stem cells; KO, knock-out; LBHI, lewy-body like hyaline inclusions; LMN, lower motor neuron; MN, motor neuron; mPrp, mouse prion promoter; MRI, magnetic resonance imaging; NCI, neuronal cytosolic inclusions; NFH, neurofilament heavy; NFL, neurofilament light; NMDA, N-methyl-D-aspartate; NMJ, neuromuscular junction; OPTN, Optineurin; PD, Parkinson’s disease; PET, positron emission tomography; PM, personalized medicine; PSP, progressive supranuclear palsy; sALS, sporadic amyotrophic lateral sclerosis; siRNA, small interfering RNA; SOD1, superoxide dismutase 1; *TARDBP* (TDP-43), TAR DNA-binding protein; TBK1, TANK-binding kinase 1; UBQLN2, Ubiliquin-2; UMN, upper motor neuron; VBM, voxel-based morphometry; VOC, volatile organic compounds
